# Trauma-focused treatments for refugee children and adolescents with PTSD: a three-arm randomized controlled trial comparing the efficacy of EMDR and KIDNET versus a waitlist control group (the KIEM study)

**DOI:** 10.1186/s13034-026-01118-0

**Published:** 2026-06-21

**Authors:** Merel E. Velu, Carlijn de Roos, Niels van der Aa, Ruud Jongedijk, Ramón Lindauer, Trudy Mooren

**Affiliations:** 1 ARQ Centrum’45, Partner in ARQ National Psychotrauma Centre, Diemen, the Netherlands; 2 Department of Clinical Psychology, Utrecht University, Utrecht, the Netherlands; 3 Department of Child and Adolescent Psychiatry, Amsterdam University Medical Centre, Amsterdam, the Netherlands; 4 Department of Oral Public Health, Academic Centre for Dentistry Amsterdam (ACTA), University of Amsterdam and VU University, Amsterdam, The Netherlands; 5 The Amsterdam Public Health research institute (APH), Amsterdam, the Netherlands

**Keywords:** Posttraumatic stress disorder, EMDR, KIDNET, Refugee, Children, Adolescents

## Abstract

**Background:**

Due to trauma and ongoing adversity, refugee minors are at high risk for mental health issues, including PTSD. This study evaluated the efficacy of Eye Movement Desensitization and Reprocessing therapy (EMDR) and Narrative Exposure Therapy for Children (KIDNET), in reducing PTSD symptoms, behavioural and emotional symptoms and improving quality of life in refugee children and adolescents in the Netherlands, compared to a waitlist control group (WL). A secondary objective was to compare the efficacy of EMDR and KIDNET.

**Methods:**

A randomized controlled trial was conducted with three arms (N = 96): EMDR (n = 32), KIDNET (n = 32), and WL (n = 32). After 8 weeks, WL participants were re-randomized to EMDR or KIDNET. Follow-ups were conducted at 1- and 3-months post-treatment. Participants were refugee minors aged 8–18 years, accompanied by a caregiver, and meeting criteria for a (partial) PTSD diagnosis. Both treatments included 8 weekly sessions and 1–4 parental guidance sessions.

**Results:**

Both EMDR (d = 1.31) and KIDNET (d = 0.94) significantly reduced clinician-rated PTSD symptom severity compared to WL. Similar results were found for child-report, but not for caregiver-report. Regarding secondary outcomes, quality of life and emotional and behavioural symptoms, comparisons of both interventions to the WL revealed small to moderate effect sizes with non-significant effects for KIDNET versus WL, and significant effects for EMDR versus WL. EMDR, compared to KIDNET, showed a significantly greater reduction in clinician-rated PTSD symptom severity (T1-T3) (d=-0.38) with no significant differences on other outcome measures. Results were achieved after an average of 6.61 sessions for EMDR and 9.10 for KIDNET. Dropout rates were 20.8% for EMDR and 10.9% for KIDNET, based on the total sample after the second randomization.

**Conclusions:**

These findings suggest that both EMDR and KIDNET are efficacious trauma-focused treatments for refugee children and adolescents.

*Trial registration* The trial was registered in the Overview of Medical Research in the Netherlands on February 2, 2014 (NL-OMON44793), amended on June 16, 2017, and re-registered on June 16, 2021 (NL-OMON22679), where one updated outcome measure and expanded eligibility criteria were documented.

## Background

By the end of 2024 an estimated 123.2 million people were forcibly displaced due to persecution, conflict, violence, or human rights violations [1], with children representing around 40% of this population. Many of these children have experienced multiple stressors, including war, violence, separation, and loss. When migrating to a new country, they frequently face ongoing adversities, such as poor living conditions, and ongoing uncertainty about asylum procedures or wellbeing of family members [2]. These adversities predispose children to the development of early-onset mental health issues, including a variety of emotional and behavioural difficulties, and posttraumatic stress disorder (PTSD), which are more prevalent among refugee children than host populations [3, 4]. A systematic review reported PTSD prevalence rates among young refugees and asylum seekers in Europe ranging from 19.0% to 52.7% [5]. In addition to exposure to adversity, refugee children face significant barriers to accessing mental health care, including stigma, unstable living conditions, language barriers, feelings of mistrust toward professionals resulting from past adverse experiences, and a lack of knowledge about available services [6–8]. When left untreated, PTSD can lead to additional mental health issues such as anxiety disorders, mood disorders, or substance use disorders, and may adversely affect children’s psychosocial functioning and personality development [4, 9]. Timely and effective trauma-focused treatment is essential to mitigate these long-term consequences. Throughout this manuscript, the term “children” refers to both children and adolescents.

Systematic reviews and meta-analyses have examined trauma-focused treatments for refugee children, demonstrating their potential to alleviate PTSD symptoms [10–12]. However, the evidence remains insufficient to inform treatment guidelines. One evidence-based trauma-focused treatment is Eye Movement Desensitization and Reprocessing therapy (EMDR), a method recommended by international guidelines for PTSD treatment [13]. Although applied and studied in predominantly Western contexts, emerging evidence supports its effectiveness and applicability for diverse cultural and ethnic groups [13]. EMDR aims to reduce emotional distress by facilitating the reprocessing of traumatic memories through dual attention tasks, such as eye movements or tactile stimulation. During EMDR, individuals recall distressing memories while simultaneously performing a secondary task, which taxes working memory and reduces the vividness and emotional intensity of the memory [14, 15]. Another evidence-based intervention is Narrative Exposure Therapy (NET), developed for individuals in crisis-affected, post-conflict and resource-limited settings, who have experienced repeated traumatic experiences. Initially designed for adults, NET was adapted for children aged approximately 8 years and older, resulting in KIDNET [16, 17]. KIDNET is successfully examined and implemented in various cultures and settings [16–21]. KIDNET involves narrative exposure to traumatic experiences within the context of the child’s life story. Using a lifeline approach, the child constructs a chronological narrative of both traumatic and positive events, while detailed narrative exposure to traumatic memories - where traumatic events are processed in their entirety rather than in isolated fragments - facilitates emotional processing and the integration of fragmented traumatic memories into a coherent autobiographical narrative [22, 23].

While existing studies have demonstrated symptom improvements in refugee children treated with EMDR or KIDNET [16–21, 24–27], significant methodological limitations, including small sample sizes, absence of control groups, lack of randomization, and not using a clinical interview for PTSD assessment highlight the necessity for more rigorous research [12, 28]. The current study was designed with a more robust methodology, to strengthen the evidence base. To our knowledge, this is the first three-arm randomized controlled trial evaluating the efficacy of two evidence-based, trauma-focused interventions, in comparison to a waitlist control group among refugee children. Additionally, this study addresses barriers to mental health care through the provision of outreach care and collaboration with intercultural mediators. The first aim was to evaluate the efficacy of EMDR and KIDNET in reducing PTSD symptom severity, behavioural and emotional symptoms, and improving quality of life in refugee children in the Netherlands, compared to a waitlist control group (WL). The second aim was to examine differences in efficacy across all outcome measures between EMDR and KIDNET.

## Methods

### Study design

A three-arm multicenter randomized controlled trial (RCT) was executed in the Netherlands. Assessments and treatment sessions were conducted at various locations across the Netherlands, including two outpatient clinics: ARQ Centrum’45, the Dutch national centre for specialized diagnostics and treatment of complex psychotrauma complaints, and I-Psy Youth and Family, a specialist centre in intercultural psychiatry. In addition, outreach care was provided, with assessors and therapists traveling to participants to conduct assessments and treatment sessions at locations close to the child’s daily environment (e.g., schools, general practitioner practices, or at home). Fifteen children (15.6%) received treatment at the two outpatient clinics, while the remaining eighty-one children (84.4%) were seen at various locations near their place of residence. These locations were determined in consultation with the child and family, considering what was most feasible and comfortable for the child, while ensuring that the setting was appropriate for therapy and provided adequate privacy. Ethics approval was obtained from the Medical Ethical Committee in Leiden, the Netherlands (reference NL40769-v0). The trial protocol has been published [29].

### Participants

Inclusion criteria were: children aged 8 up to and including 18 years old, accompanied by at least one caregiver, who had applied for asylum in the Netherlands or had been residing there since January 2015 or later. Participants were required to meet the criteria for a partial or full PTSD diagnosis assessed with the Clinician Administered PTSD Scale for DSM5 - Child/Adolescent Version (CAPS-CA-5) [30, 31]. Partial PTSD was defined as meeting the criteria for three of the four symptom clusters or having one symptom from each of the four clusters, in addition to fulfilling criteria A, F, and G. Exclusion criteria were: an estimated intelligence level below 80, acute interfering psychiatric disorders in need for treatment first (e.g., acute suicidality, brain damage, acute threat of deportation or relocation abroad during the intervention period, current anti-epileptic and neuro-epileptic medication use, or severe substance abuse). Participants were recruited through various channels, including schools, asylum centres, international transitional classes for students aged 12–18 with limited Dutch proficiency, general practitioners, youth and family centres, and social media posts, sometimes using psychoeducational videos in the participants’ native languages. Simultaneously, recruitment took place at the two outpatient clinics mentioned above. Eritrean and Syrian intercultural mediators assisted with recruitment and intake, offering translation support, and building trust by sharing their cultural backgrounds, explaining the study and addressing mental health stigma.

### Procedure

After obtaining written informed consent, baseline data (T1) were collected, and eligible participants were assigned an unique number and matched with an available therapist. Hereafter, participants were randomized to treatment condition, ensuring balanced allocation across therapists. All therapists were trained in and delivered both EMDR and KIDNET. An independent research methodologist from ARQ Centrum’45 (NvdA) used R to generate a random sequence for the three treatment arms (EMDR, KIDNET, and waitlist), applying a randomized block design with a 1:1:1 allocation ratio and variable block sizes. A second random sequence (EMDR vs. KIDNET) was generated for participants initially allocated to the waitlist. After randomization, the research methodologist notified the research coordinator of the allocated condition, who then notified the therapists. Treatment began within four weeks for participants in the active conditions, with assessments at baseline, 1- and 3-months post-treatment. Waitlist participants were assessed at baseline, and after eight weeks of waiting. Hereafter, WL participants were re-allocated to either EMDR or KIDNET and reassessed 1- and 3-months post-treatment. Participants received a €15 gift card for each post- and follow-up assessment. No trauma-focused treatment was provided between the last session and follow-up assessments. Due to the nature of the interventions, it was not possible to blind therapists nor participants for the treatment allocation. The independent assessors remained blinded to the specific treatment allocation (EMDR or KIDNET), although they were aware of the participant’s assignment to either the waitlist or a treatment condition.

EMDR followed the Dutch translation of Shapiro’s eight-phase protocol for children [14, 32], while KIDNET used the Dutch adaptation of the NET protocol for children [22, 23]. The initial session of both treatments included psychoeducation and an explanation of the selected therapeutic method. In the first EMDR session, emotionally charged target memories were identified as part of the case conceptualisation process. Subsequent sessions then focused on reducing the emotional distress associated with these memories. In KIDNET, the child created a lifeline depicting both traumatic and positive life events during the initial session. These memories were then processed chronologically through narrative exposure in the sessions that followed. Both interventions consisted of up to eight weekly sessions, each lasting 75 min, including the initial case conceptualisation session. For EMDR, early termination was possible if target memories no longer evoked tension or if symptoms resolved. For KIDNET, the lifeline was always fully processed, with early termination only possible when the chronological processing of the lifeline had already been completed before the eighth session. If additional sessions were needed, this had to be approved during supervision (see below). Caregivers received one to four parental psychoeducation and guidance sessions, focusing on supporting the child. It was possible for therapists to collaborate with a Syrian or Eritrean cultural mediator during assessments or treatment. Additionally, a telephone interpreter service was always available, providing interpretation in all required languages.

The interventions were delivered by nine therapists, and ten assessors conducted the assessments. All therapists were certified clinical psychologists who completed a four-day basic Dutch accredited training in NET, and at least one supervised KIDNET treatment. Additionally, each therapist had completed a 5.5-day training for EMDR Europe Level 1 and an additional 4.5-day training for Level 2 including competency verification and showing video recordings of sessions. With participant consent, all sessions were video- or audio-recorded. To ensure treatment adherence, therapists received supervision every six weeks (1.5 h per session) from accredited EMDR and KIDNET supervisors, based on session recordings. In addition, to monitor treatment fidelity, 20% of EMDR sessions, and 20% of KIDNET sessions were reviewed. Treatment fidelity was rated as excellent in all reviewed sessions, with a mean competency score of 97.1% for both EMDR and KIDNET.

### Primary outcomes

- PTSD diagnosis and symptom severity assessed by the CAPS-CA-5, a structured clinical interview aligned with DSM-5 [30, 31]. At baseline, the LEC-5 was administered to identify the event causing the most distress for the child. At each timepoint (T1–T3), the CAPS-CA-5 was administered based on the traumatic event that participants identified as currently most stressful [31]. The CAPS-CA-5 includes 30 items and four subscales: intrusion, avoidance, negative cognitions and mood, and arousal and reactivity symptoms. The total severity score (0–80) is the sum of all items, with higher scores indicating greater severity of PTSD. The reliability of the CAPS-CA-5 was α = 0.80 at baseline.
- PTSD severity evaluated using the CRIES-13, a brief DSM-IV based 13-item questionnaire, with both child- and caregiver report [33, 34]. An elevated score reflects more severe symptoms. At baseline, the reliability of the CRIES-13 was α = 0.75 for child-report and α = 0.83 for caregiver-report. Baseline data (T1) of the CRIES-13 child version were missing for the first eight participants, as the CRIES-8 was administered instead. At the time of the study, a PTSS screener based on the DSM-5 was not available in the Netherlands.

### Secondary outcomes

- Behavioural and emotional symptoms using the total difficulties score of the Strengths and Difficulties Questionnaire (SDQ) [35]. An elevated score is indicative of more severe symptoms. The SDQ child version was administered to participants aged 11 years and older, while the caregiver version was used for participants of all ages. At the baseline assessment (T1), 74 participants (77.1% of the sample) were aged 11 years or older. The internal consistency of the SDQ total score was α = 0.53 for the child-report version and α = 0.52 for the parent-report version.
- Quality of life using the child version of the KIDSCREEN-27c [36]. A higher score reflects a better quality of life. The reliability of the KIDSCREEN-27c was α = 0.83.

All outcomes were measured at each timepoint. The CRIES-13 and SDQ (child and parent versions) were translated and back-translated by bilingual interpreters into Tigrinya and the CRIES-13 parent version was translated into Arabic. The CRIES-13 and the SDQ were available online in several languages, with official translations. For other languages, the assessments were completed in Dutch, with (telephone) interpretation provided.

### Statistical analysis

For the first comparison (EMDR and KIDNET vs. WL), a priori sample size calculation in G*Power [37] indicated that 78 participants (26 per group) were needed to detect a modest effect size (f = 0.20, α = 0.05, power = 0.80). Additionally, 70 participants (35 per group) were required to detect a small effect size (f = 0.17) when comparing EMDR to KIDNET across three time points using the same assumptions. We based our design on the larger required sample size (*n* = 78). To allow for 20% attrition and ensure balanced group sizes, the sample size was increased to 96 participants. Data from WL participants secondly randomized to treatment (*n* = 32) were only included in the EMDR vs. KIDNET comparison, resulting in group sizes of 48 participants per active treatment condition.

Between-group comparisons on primary and secondary outcomes were carried out using linear mixed models (LMMs), using SPSS version 27. LMM was selected instead of GLM due to its ability to handle missing data flexibly under the assumption that the data are missing at random, allowing inclusion of all available data without excluding incomplete cases. As 81 participants completed treatment, no separate completer analyses were performed, and analyses were conducted following the intention-to-treat principle. First, EMDR (*n* = 32) and KIDNET (*n* = 32) were each compared to WL (*n* = 32), focusing on pre-treatment (T1) to post-treatment (T2) changes. Although the hypothesis for this comparison is directional, specifically that TFT (EMDR and KIDNET) would show greater improvement in all outcome measures compared to WL, two-sided testing was chosen instead of one-sided testing to reduce the risk of increased Type 1 errors from multiple comparisons. Due to their conservativeness and the expected increased risk of Type II error in our medium sample size, strict multiple comparison corrections (e.g., Bonferroni) were not used [38, 39]. Second, EMDR (*n* = 48) was compared to KIDNET (*n* = 48), examining changes in posttraumatic stress symptoms and related outcomes over the course of treatment from pre-treatment (T1) to post-treatment (T2), and from pre-treatment (T1) to 3-month follow-up (T3). Analyses included fixed effects for time, and condition (EMDR, KIDNET, or waitlist), as well as their interaction (group x time). We specified the LMM with the WL as the reference category. Random intercepts were modelled for participants to account for potential individual differences at baseline. The assumption of normality was visually assessed. For each outcome measure, separate LMM’s were ran. Effect sizes, Cohen’s d, were calculated by dividing the mean difference, derived from the mixed model analyses, by the pooled standard deviation. The pooled SD was calculated as the square root of the sample size (N), multiplied by the standard error of the mean difference (SE). Baseline demographic differences between conditions were examined using one-way ANOVAs for continuous variables and chi-square tests for categorical variables.

The trial was initially registered with the Overview of Medical Research in the Netherlands on February 2, 2014 (NL-OMON44793). Following this 2014 registration, an amendment containing several changes was submitted and approved on June 16, 2017. After this 2017 amendment, three further protocol modifications were implemented: (1) inclusion of children with residence permits (2), inclusion of participants meeting criteria for partial PTSD, and (3) the use of the CRIES-13 instead of the CRIES-8. These modifications were documented in a new registration on June 16, 2021 (NL-OMON22679). Although the trial was registered in 2014, recruitment commenced in 2018 after additional funding was obtained to establish the necessary research infrastructure, including study coordination and recruitment procedures.

## Results

**Fig. 1:**
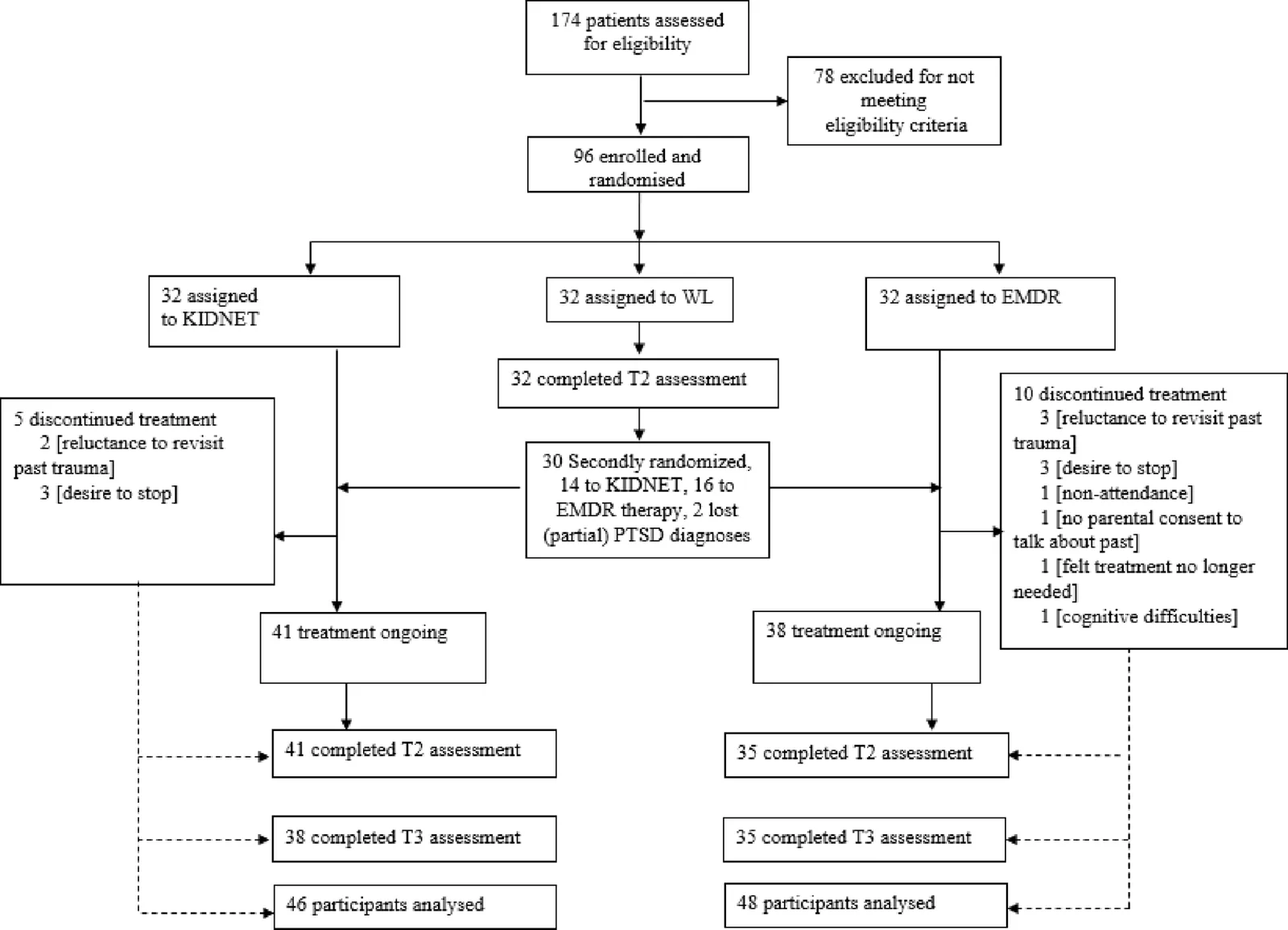
Trial Profile

Participants were recruited between February 15, 2018, and June 6, 2023. Figure 1 presents the trial profile. Numbers of completed assessments reflect available CAPS-CA-5 data at each time point. Following the WL period, two participants reported spontaneous recovery and were not re-allocated into EMDR or KIDNET. A total of 15 of 96 participants (15.6%) discontinued treatment in the combined treatment sample, 10 of 48 (20.8%) in the EMDR group, and 5 of 46 (10.9%) in the KIDNET group.

Table 1 provides information about age, gender, country of origin, residence status, time in the Netherlands and the most distressing traumatic event. No baseline differences across treatment conditions on any demographic characteristics were found.

**Table 1:** Demographic characteristics of study participants at baseline

Variable	EMDR (*n* = 32)	KIDNET (*n* = 32)	WL (*n* = 32)	Total sample (*n* = 96)
Age in years	13.31 (2.9)	13.14 (3.5)	14.13 (3.2)	13.52 (3.2)
Sex				
Female	12 (37.5)	12 (37.5)	14 (43.8)	38 (39.6)
Male	20 (62.5)	20 (62.5)	18 (56.3)	58 (60.4)
Country of origin				
Syria	21 (65.6)	20 (62.5)	23 (71.9)	64 (66.7)
Eritrea	4 (12.5)	1 (3.1)	··	5 (5.2)
Afghanistan	··	4 (12.5)	2 (6.3)	6 (6.3)
Turkey	1 (3.1)	1 (3.1)	2 (6.3)	4 (4.2)
Iran	.	2 (6.3)	1 (3.1)	3 (3.1)
Iraq	2 (6.3)		1 (3.1)	3 (3.1)
Armenia	2 (6.3)	··	1 (3.1)	3 (3.1)
Other^a^	2 (6.3)	4 (12.5)	··	8 (25.0)
Status				
Awaiting	4 (12.5)	7 (21.9)	6 (18.8)	17 (17.7)
Residence permit	28 (87.5)	25 (78.1)	26 (81.3)	79 (82.3)
Time in Netherlands in months^b^	31.37 (23.0)	29.71 (23.7)	28.26 (18.4)	29.69 (21.4)
Most distressing traumatic event^c^				
War-related violence^d^	21 (65.6)	21 (65.5)	25 (78.1)	67 (69.8)
Family members died due to war violence	5 (15.6)	1 (3.1)	2 (6.3)	8 (8.3)
Traumatic sea crossing during the flight	1 (3.1)	4 (12.5)	2 (6.3)	7 (7.3)
Been imprisoned or kidnapped	2 (6.3)	1 (3.1)	2 (6.3)	5 (5.2)
Sexual abuse	6 (18.8)	4 (12.5)	2 (6.3)	12 (12.5)
(witnessed) physical abuse in family	5 (15.6)	2 (6.3)	3 (9.4)	10 (10.4)
Other ^e^	3 (9.4)	5 (15.6)	5 (15.6)	13 (13.5)

Data are presented as n (%) or mean (SD). ^a^Other includes Bahrain, Ivory Coast, Yemen, Libia, Marocco, Congo, Ukraine, Uganda. ^b^Due to missing data, time in Netherlands data was available for 69 participants. When only the month and year of arrival were known, the 15th of that month was selected. ^c^measured with the LEC, 11 participants identified two events as the most distressing, both of which were included, resulting in a total number higher than 96. ^d^The numbers shown for war-related violence also include: family members who died due to war violence, traumatic sea crossings during flight, and experiences of imprisonment or kidnapping. ^e^ Other includes witnessing the arrest of a parent in the Netherlands, armed robbery, accident, suicide attempt or being attacked or beaten

Table 2 presents the results of the comparison between EMDR and KIDNET versus WL.

**Table 2:** Summary statistics and results from LMM analysis of primary and secondary outcomes for EMDR therapy and KIDNET vs. WL comparison

	Descriptives						Mixed model analysis EMDR therapy vs. WL		Effect size	Mixed model analysis KIDNET vs. WL		Effect size
	EMDR Mean (SD)	*N*	KIDNET Mean (SD)	*N*	WL Mean (SD)	*N*	Difference (95% CI)	*p* value	Cohen’s d (95% CI)	Difference (95% CI)	*p* value	Cohen’s d (95% CI)
CAPS-CA-5												
T1	28.84 (10.88)	32	31.40 (10.97)	32	28.72 (10.88)	32	··	··	··	··	··	··
T2	6.96 (10.23)	24	14.80 (10.64)	29	24.13 (10.88)	32	17.29 (12.64 to 21.93)	< 0.0001*	1.31 (0.96 to 1.66)	12.00 (7.52 to 16.49)	< 0.0001*	0.94 (0.59 to 1.29)
CRIES-13 child												
T1	39.04 (14.09)	28	39.80 (14.14)	30	38.87 (14.13)	28	··	··	··	··	··	··
T2	14.36 (13.84)	23	21.05 (14.04)	28	34.37 (14.03)	26	20.19 (11.78 to 28.60)	< 0.0001*	0.90 (0.53 to 1.28)	14.26 (6.23 to 22.29)	0.0007*	0.65 (0.28 to 1.01)
SDQ child												
T1	17.24 (6.42)	25	19.89 (6.40)	22	16.00 (6.41)	25	··	··	··	··	··	··
T2	11.68 (6.30)	18	15.34 (6.40)	22	15.46 (6.39)	24	5.03 (0.60 to 9.45)	0.0265*	0.45 (0.05 to 0.85)	4.01 (-0.30 to 8.33)	0.0678	0.37 (-0.03 to 0.77)
KIDSCREEN-27												
T1	97.39 (19.14)	26	93.36 (18.63)	24	96.53 (18.68)	26	··	··	··	··	··	··
T2	109.98 (18.57)	22	103.58 (19.02)	27	98.18 (18.68)	26	-10.95 (-20.94 to -0.97)	0.0321*	-0.43 (-0.82 to -0.04)	-8.58 (-18.43 to 1.27)	0.0868	-0.33 (-0.72 to 0.05)
CRIES-13 caregiver												
T1	32.90 (14.98)	23	40.91 (15.05)	24	33.33 (14.95)	23	··	··	··	··	··	··
T2	21.22 (14.74)	17	30.27 (14.84)	19	29.04 (13.92)	18	7.39 (-3.95 to 18.73)	0.1971	0.27 (-0.15 to 0.69)	6.35 (-4.63 to 17.33)	0.2516	0.24 (-0.17 to 0.65)
SDQ caregiver												
T1	16.04 (6.66)	27	17.64 (6.59)	23	16.00 (6.65)	26	··	··	··	··	··	··
T2	11.17 (6.10)	17	13.91 (6.34)	19	15.07 (6.19)	18	3.93 (0.27 to 7.60)	0.0359*	0.42 (0.03 to 0.81)	2.79 (-0.83 to 6.42)	0.1279	0.30 (-0.09 to 0.70)

**Fig. 2:**
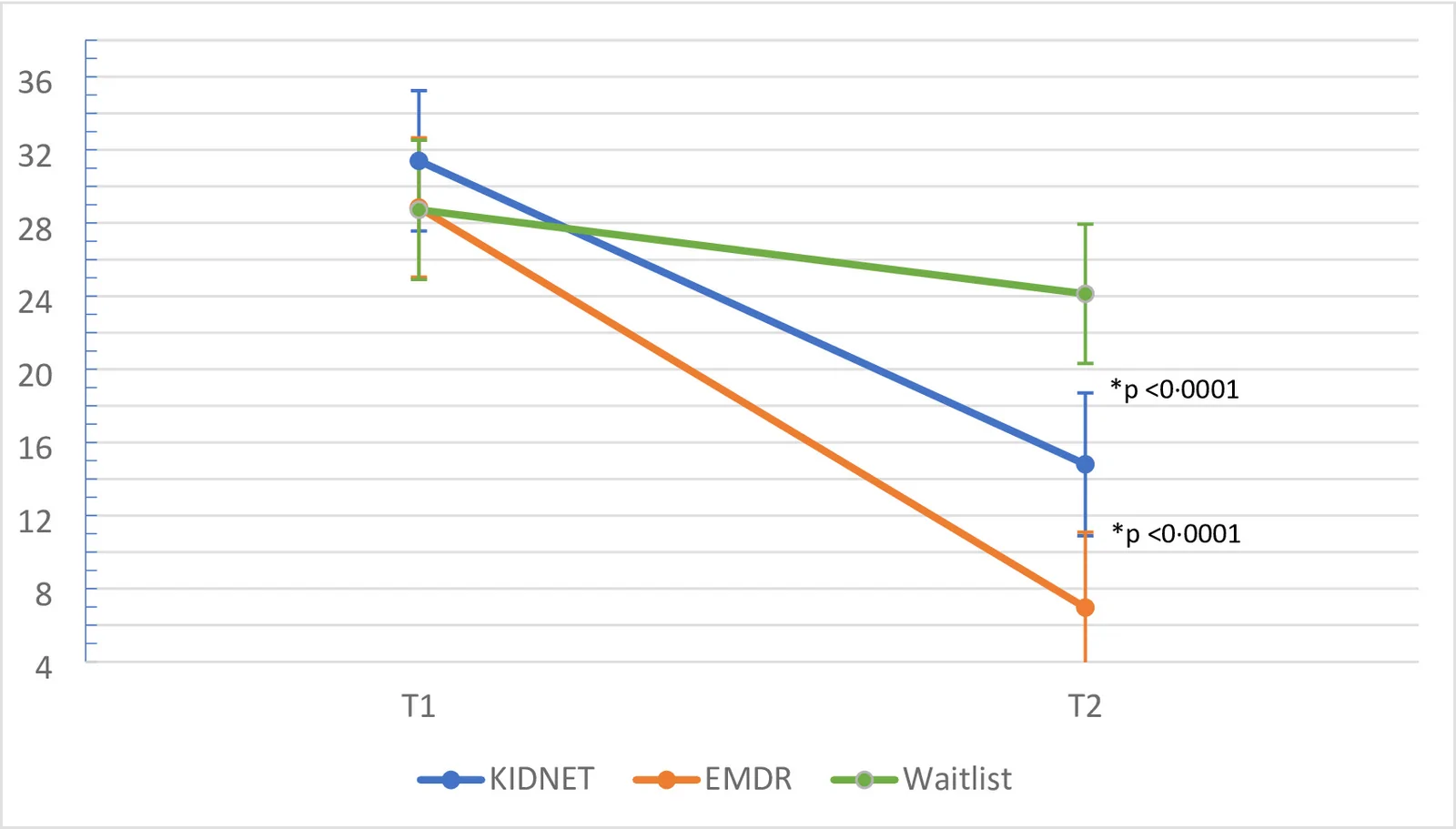
Mean PTSD Symptom Severity (CAPS-CA-5) and 95% Confidence Intervals at T1-T2 for EMDR, KIDNET and WL.

Both EMDR (difference = 17.29, 95% CI:12.64–21.93, *p* < 0·0001, *d* = 1.31) and KIDNET (difference = 12.00, 95% CI:7.52–16.49, *p* < 0·0001, *d* = 0.94) demonstrated significant larger reductions in CAPS-CA-5 scores compared to WL with large effect sizes. See Fig. 2 for the mean CAPS-CA-5 scores, along with 95% confidence intervals at baseline (T1) and post-treatment (T2) for the three conditions. The y-axis is limited to the observed range of CAPS-CA-5 scores in this study and does not represent the full possible scale In addition, both interventions revealed significant larger reductions in self-reported PTSD symptoms (CRIES-13) with a medium to large effect size for KIDNET vs. WL (*d* = 0.65) and a large effect size for EMDR vs. WL (*d* = 0.90). Change in caregiver-reported PTSD symptoms (CRIES-13) for either intervention was not significantly different compared to WL. Regarding the secondary outcomes, EMDR revealed significant larger reductions in behavioural and emotional symptoms (SDQ child- and caregiver report), and improvement of quality of life (KIDSCREEN-27c), relative to WL, with small to moderate effect sizes. For KIDNET the change in SDQ scores (child- and caregiver report) and KIDSCREEN-27c scores was not statistically significant different from WL, with small to moderate effect sizes.

The results of the comparison between EMDR (*n* = 48) and KIDNET (*n* = 46) are summarized in Table 3. To examine whether participants entering treatment after the WL period differed from those directly assigned to EMDR or KIDNET, we compared baseline PTSD symptom severity (CAPS-CA-5) at treatment start using a one-way ANOVA. No significant differences were found between groups. EMDR demonstrated significantly greater reductions in CAPS-CA-5 scores compared to KIDNET from T1 to T2 (difference=-7·05, 95% CI:-11.86 to -2.23, *p* = 0·0043, *d* = 0.42) and from T1 to T3 (difference=-6·49, 95% CI:-11·42 to -1·55, *p* = 0·0106, *d*=-0·38).

**Fig. 3:**
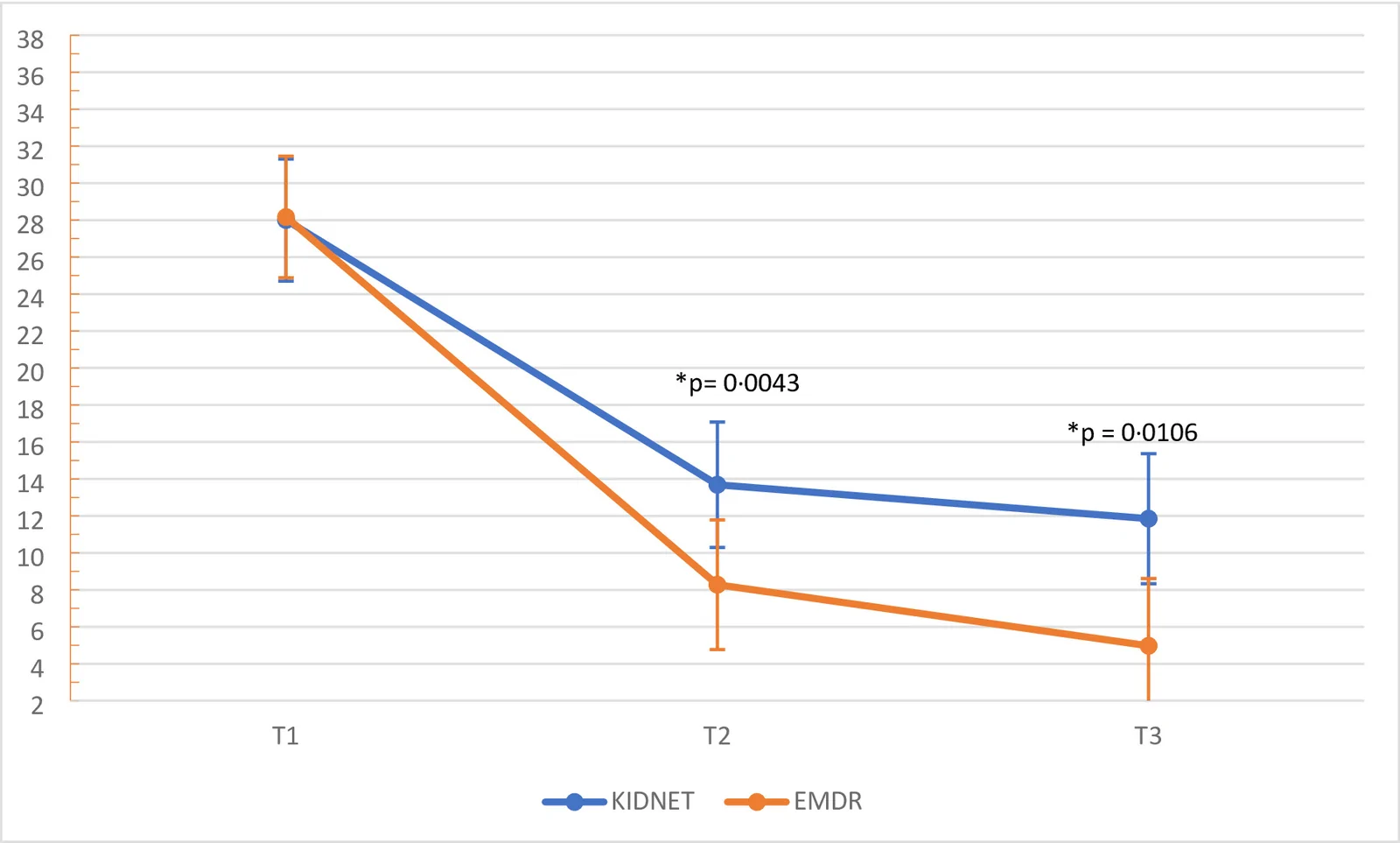
Mean PTSD Symptom Severity (CAPS-CA-5) with 95% Confidence Intervals at T1-T3 for EMDR and KIDNET.

See Fig. 3 for the mean levels of PTSD symptom severity (CAPS-CA-5) scores for both the KIDNET (*n* = 46) and EMDR (*n* = 48) condition at three time points. No significant differences between EMDR and KIDNET were found for any of the other outcomes.

Table 4 presents the number of participants meeting the criteria for a (partial) PTSD diagnosis at Pretreatment (T1) and Post Treatment (T2), comparing the EMDR and KIDNET groups with the WL group. At T2, 5 participants (15.6%) in the EMDR group, 12 participants (37.5%) in the KIDNET group and 30 participants (93.8%) in the WL group satisfied the criteria for a (partial) PTSD diagnoses.

**Table 3:** Summary statistics and results from LMM analysis for primary and secondary outcomes for EMDR therapy vs. KIDNET comparison

	Descriptives				Mixed model analysis		Effect size
	EMDR therapy Mean (SD)	*N*	KIDNET Mean (SD)	*N*	Difference between T1-T2 and between T1-T3 (95% CI)	*p* value	ES between T1-T2 and between T1-T3 (Cohen’s d 95% CI)
CAPS-CA-5							
T1	28.17 (12.73)	48	28.01 (12.20)	46			
T2	8.28 (5.92)	35	13.69 (6.40)	41	-7.05 (-11.86 to -2.23)	0.0043*	-0.42 (-0.70 to -0.13)
T3	4.97 (10.87)	35	11.85 (10.25)	38	-6.49 (-11.42 to -1.55)	0.0106*	-0.38 (-0.09 to -0.67)
CRIES-13 child							
T1	38.70 (14.54)	41	37.08 (14.58)	43			
T2	20.59 (13.98)	34	20.91 (14.34)	40	-6.16 (-13.34 to 1.07)	0.0915	-0.26 (-0.56 to 0.04)
T3	16.05 (14.36)	35	20.59 (14.37)	38	-4·80 (-11.97 to 2.37)	0.1869	-0.20 (-0.51 to 0.10)
SDQ child							
T1	16.62 (6.74)	37	18.33 (6.75)	34			
T2	11.25 (6.39)	28	14.62 (6.66)	32	-1.87 (-5.43 to 1.69)	0.2995	-0.17 (-0.50 to 0.16)
T3	10.71 (6.55)	29	14.28 (6.57)	28	-2.04 (-5.75 to 1.67)	0.2759	-0.18 (-0.51 to 0.15)
KIDSCREEN-27							
T1	96.66 (19.91)	39	95.94 (19.38)	37			
T2	107.92 (19.11)	32	104.87 (19.09)	35	6.98 (-2.19 to 16.14)	0.1336	0.24 (-0.08 to 0.56)
T3	111.59 (19.17)	31	103.89 (19.11)	34	6.59 (-2.95 to 16.13)	0.1723	0.22 (-0.10 to 0.54)
CRIES-13 caregiver							
T1	32.18 (14.91)	32	36.64 (14.94)	33			
T2	20.34 (14.03)	23	26.17 (14.28)	26	0.14 (-9.37 to 9.65)	0.9762	0.01 (-0.34 to 0.35)
T3	18.81 (14.79)	28	23.14 (14.76)	28	-1.62 (-10.97 to 7.73)	0.7303	-0.06 (-0.41 to 0.29)
SDQ caregiver							
T1	15.56 (6.69)	36	16.78 (6.60)	32			
T2	11.14 (6.12)	23	12.67 (6.32)	26	-1.53 (-4.84 to 1.79)	0.3604	-0.15 (-0.49 to 0.18)
T3	9.67 (6.40)	28	12.42 (6.45)	28	-1.82 (-5.11 to 1.47)	0.2723	-0.18 (-0.52 to 0.15)

**Table 4:** Number (%) of participants meeting (Partial) PTSD diagnoses at T1-T2, for the EMDR therapy and KIDNET vs. WL comparison

	T1			T2		
	EMDR	KIDNET	WL	EMDR	KIDNET	WL
PTSD diagnoses	24 (75.0)	26 (81.3)	23 (71.9)	0 (0.0)	9 (28.1)	15 (46.9)
Partial PTSD diagnoses	8 (25.0)	6 (18.8)	9 (28.1)	5 (15.6)	3 (9.4)	15 (46.9)
No (partial) PTSD diagnoses	…	…	…	20 (62.5)	17 (53.1)	2 (6.3)

Data is in n (%). Due to missing data, the number of available observations vary across time points. Percentages are based on the full sample (*n* = 32 per group)

Table 5 presents the number of participants fulfilling a (partial) PTSD diagnosis at Pretreatment (T1), Post Treatment (T2) and Three-Month Follow-up (T3), comparing the EMDR and KIDNET groups. At T3, 5 participants (10.4%) in the EMDR group and 11 participants (23.9%) in the KIDNET group met the criteria for a (partial) PTSD diagnosis.

**Table 5:** Number (%) of Participants Meeting (Partial) PTSD Diagnoses at T1-T3, for the EMDR therapy vs. KIDNET Comparison

	T1		T2		T3	
	EMDR	KIDNET	EMDR	KIDNET	EMDR	KIDNET
PTSD diagnosis	33 (68.8)	32 (69.6)	2 (4.2)	12 (26.1)	5 (10.4)	4 (8.7)
Partial PTSD diagnosis	15 (31.3)	14 (30.4)	6 (16.2)	4 (9.8)	0 (0.0)	7 (15.2)
No (partial) PTSD diagnoses	…	…	29 (60.4)	25 (54.3)	32 (66.7)	28 (60.9)

Data is in n (%). Due to missing data, the number of available observations vary across time points. Percentages for EMDR are based on a total sample of *n* = 48 and for KIDNET on *n* = 46

Completers in the EMDR condition attended an average of 6.61 sessions (SD = 1.99), and caregivers from 37 participants (77.1%) participated in parental guidance sessions with an average of 2.57 (SD = 1.07). In the KIDNET condition, completers received an average of 9.10 sessions (SD = 3.05), with caregivers from 38 participants (82.6%) participating in an average of 2.71 (SD = 1.11) parental guidance sessions. Additionally, nine participants in the EMDR condition received an average of 1.78 extra sessions focused on current stressors, safety concerns, motivation for treatment, and supportive contact. One participant in this group also received family therapy between the baseline- and three months follow-up. In the KIDNET condition, four participants attended an average of 2.50 additional sessions addressing current stressors, complaints, and emotion regulation. One participant also received additional treatment between the baseline- and three months follow-up, which included physical and movement therapy, and supportive conversations. No other treatment was provided between the baseline assessment and follow-up assessments.

## Discussion

The present study is the first three-arm randomized controlled trial demonstrating the efficacy of two evidence-based trauma-focused interventions (EMDR and KIDNET) compared to a WL among 96 refugee children aged 8–18. Both interventions led to significantly greater reductions in clinician-reported and self-reported PTSD symptom severity with medium to large effect sizes compared to WL, suggesting their use as efficacious trauma-focused treatments for this population. EMDR, compared to KIDNET, resulted in a significantly greater reduction in CAPS-CA-5 scores from baseline to both one-month and three-month follow-up assessments, while no differences were found on other outcome measures.

Both the clinician-reported and child-reported PTSD severity outcomes in this trial align with previous research and support our hypotheses that both EMDR and KIDNET are efficacious compared to WL for refugee children exposed to traumatic events [12]. Regarding quality of life and emotional and behavioural symptoms, comparisons of both interventions to the WL revealed small to moderate effect sizes with non-significant effects for KIDNET versus WL, and significant effects for EMDR versus WL. These effect sizes indicate a noticeable but modest impact. Both trauma-focused treatments demonstrate their strongest effect on trauma specific symptoms, reflecting the fact that the intervention was deliberately designed and tailored to directly address these symptoms as the primary therapeutic target. In the light of the common persistent stressors in the daily life of refugee children, the presence of emotional and behavioural symptoms and impairments in quality of life may be understandable.

Contrary to our expectations, caregiver-reported outcomes did not show significant improvements in PTSD symptom severity (CRIES-13) for either intervention or in SDQ scores for KIDNET. The discrepancy between caregiver- and child-report outcomes may be attributed to small sample sizes for caregiver reported outcomes, which limited the statistical power to detect significant changes. An alternative explanation is that many children in this sample have parents with post-traumatic stress symptoms, who can be less sensitive towards their children due to their own psychological distress [41]. As a result, changes in symptoms may be less noticed by caregivers.

Comparing EMDR to KIDNET, this study identified a significant greater reduction in PTSD symptom severity for EMDR, as measured with the CAPS-CA-5, from baseline to one-month and three-month follow-up assessment. No differences between the two interventions were found on other outcome measures. Notably, the outcomes in this study were achieved in an average of 6.61 sessions with EMDR, and 9.10 sessions with KIDNET, indicating that EMDR treatment was typically shorter, ending when target memories no longer elicited tension or when symptoms had resolved. KIDNET, however, always involves completion of the full lifeline, regardless of symptom reductions, which inherently required more time. The comparison between EMDR and KIDNET has not been previously investigated. Differences in their underlying mechanisms may help explain the variation in PTSD symptom reduction and the speed of recovery. KIDNET follows a chronological life timeline, helping children gradually integrate traumatic memories into their broader life narrative, a process that may take longer to yield results. In contrast, EMDR directly targets specific traumatic experiences, prioritising those with the strongest emotional charge and simultaneously engaging the child in a memory taxation task. This more focused and largely non-verbal approach in EMDR may facilitate faster emotional processing of trauma. Previous studies support this interpretation, indicating that EMDR is associated with a rapid reduction in PTSD symptoms, shortly after treatment, while the effects of (KID)NET tend to become more potent over time, reflecting its longer-term impact [12, 42, 43].

The dropout rate in the EMDR condition (20.8%) was considerably higher than in the KIDNET condition (10.9%). This is in line with a recent meta-analysis, which found that narrative exposure therapy was associated with a lower risk of dropout when considered alongside other psychotherapies [12, 44]. One possible explanation is that the structured nature of EMDR and the rapid engagement in distressing memories, may be more challenging for children who typically avoid distressing stimuli. Furthermore, the life-course perspective and positive life events embedded in KIDNET may enhance adherence. All analyses were conducted using an intention-to-treat approach with LMM, which incorporate all available data and reduce the risk of attrition bias due to differential dropout between conditions.

This study benefitted from several strengths including utilizing a randomized controlled design with broad inclusion criteria, a relatively large sample in comparison to previous studies with this population including both children and adolescents, blinded assessors for treatment condition, multi-informant diagnostic assessments, a clinical diagnostic interview for PTSD in children (CAPS-CA-5) and manualized treatments with independent fidelity checks. Another strength was the possibility of an outreach-based approach, and collaboration with intercultural mediators, which likely improved accessibility for refugee children who often face barriers to mental health care [6–8]. The total low drop-out rate (15.6%) underscores the effectiveness of this approach in maintaining participant engagement, especially within an often hard-to-reach population.

Participants were recruited through various channels. Although combining outreach and clinic recruitment may have resulted in a heterogeneous sample with varying symptom severity, the observation of significant effects within this varied sample underscores the generalizability of the findings. Moreover, the inclusion of participants with a partial PTSD diagnosis - common in clinical practice – further enhances the ecological validity and generalizability of the results. It is well established that partial PTSD can have a serious impact on psychosocial functioning and does require clinical attention [45]. While baseline PTSD symptom severity scores may be relatively low, our results were significant. In addition, most participants had a residence permit, which may reduce some post-migration stress and could limit the generalizability of findings. However, this does not eliminate other post-migration stressors such as concerns about family members remaining in conflict zones, housing instability, discrimination, and acculturation stress, all of which may impact functioning and treatment response [2].

Several limitations should be noted. First, although assessors were blinded to the specific trauma treatment (EMDR or KIDNET), assessors were not blinded to whether participants were in an active treatment or WL condition, potentially introducing bias. This was due to differences in the time interval between T1 and T2 across conditions (a fixed 8-week interval for WL versus a longer interval for active treatment), which could allow assessors to deduce allocation based on timing. Due to logistical constraints, it was not feasible for each measurement to be conducted by a different assessor. In addition, this approach ensured continuity and helped build trust, an essential factor for this population, as refugee families may experience mistrust toward mental health professionals [8]. Using the same assessor (and the same intercultural mediator, if applicable) to conduct all measurements for each participant was therefore considered important to support engagement and facilitate openness during assessments. Second, the CRIES-13 is a screening tool based on the DSM-IV, rather than the DSM-5, which may limit its ability to fully capture the symptoms according to the current diagnostic criteria. Third, a longer period between T1 and T2 in the treatment conditions compared to the fixed 8-weeks period between T1 and T2 in the WL condition, may have introduced a temporal confound. Due to the urgency of providing treatment, the duration of the WL was kept as short as possible for ethical reasons. Yet, the difference in timing of assessments does not put the outcomes on equal footing and creates a risk of biasing results. This could favour the active treatments, as they had additional weeks to demonstrate therapeutic benefit. However, Table 2; Fig. 2 show a minimal decrease in PTSD symptom severity (CAPS-CA-5) for the WL compared to the active treatment conditions. In addition, the decrease in the WL group (4.59 points) is considerable smaller than the suggested cut-off for clinical significant change on the CAPS-5 (i.e., a difference of at least 12–13 points) [40]. Although it cannot be ruled out, it is unlikely that the decrease in PTSD symptom severity in the WL group would be comparable to the active treatment conditions if the T1-T2 interval were equivalent. Fourth, the internal consistency of the total difficulties score of the SDQ (child- and caregiver report) is relatively low, indicating limited reliability in this sample. Previous concerns have been raised regarding the use of the total difficulties score of the SDQ [46]. This should be considered when interpreting SDQ results. Fifth, the three-month follow-up limits conclusions regarding the long-term treatment effects. Given that refugee children face prolonged and cumulative stressors, assessing the durability of treatment benefits over extended periods is crucial. Finally, the generalizability of findings beyond the predominantly Syrian sample is uncertain, although the underlying mechanisms of PTSD are unlikely to differ substantially across populations [47].

Future studies should investigate factors affecting treatment efficacy, including individual differences, to optimize outcomes. Additionally, future studies should explore the applicability of these interventions in low- and middle-income countries, where the majority of refugees reside, and where mental health resources are often scarce [48]. Comparing the scalability and cost-effectiveness of EMDR and KIDNET in resource-limited settings could provide valuable insights for implementation. The resource requirements for each intervention may also impact their feasibility in these contexts. EMDR requires specialized therapist training, whereas KIDNET may be more adaptable for delivery by paraprofessionals, making it a potentially more viable option in low-resource settings [22]. In current clinical practice, the choice between EMDR and KIDNET should be guided by factors such as availability, therapist training, and patient preference. Given the absence of consistent differences across outcomes and the limited number of direct comparative studies, the present findings do not support firm recommendations regarding differential treatment selection or sequencing. Future research could further explore the optimal therapeutic strategy for this population.

## Conclusions

Findings of this RCT suggest that both EMDR and KIDNET are efficacious trauma-focused treatments for refugee children and confirm that it is feasible to effectively reach and treat this population, thereby mitigating the potential long-term consequences of PTSD. These findings are especially relevant given the ongoing stressors experienced by refugee populations, underscoring that efficacious trauma-focused treatment can be successfully provided despite persistent challenges.

## Data Availability

The datasets generated and analysed during the current study are not publicly available due to privacy and ethical restrictions. The deidentified dataset that support the findings of this study will be available from the corresponding author on reasonable request after 2 years from trial publication.

## Abbreviations

PTSD: Posttraumatic stress disorder
EMDR: Eye Movement Desensitization and Reprocessing therapy
KIDNET: Narrative Exposure Therapy for children
NET: Narrative Exposure Therapy
WL: Waitlist control group
RCT: Randomized controlled trial
CAPS-CA-5: Clinician Administered PTSD Scale for DSM5 - Child/Adolescent Version
SDQ: Strengths and Difficulties Questionnaire
LMMs: Linear mixed models
